# Mycorrhizas influence functional traits of two tallgrass prairie species

**DOI:** 10.1002/ece3.2129

**Published:** 2016-05-17

**Authors:** Joanna Weremijewicz, Kotaro Seto

**Affiliations:** ^1^ Department of BiologyUniversity of MiamiP.O. Box 249118Coral GablesFlorida33124‐0421

**Keywords:** *Andropogon gerardii*, arbuscular mycorrhizal fungi, *Elymus canadensis*, functional traits, mycorrhiza dependence, mycorrhiza responsiveness, plant–soil (below‐ground) interactions

## Abstract

Over the past decade, functional traits that influence plant performance and thus, population, community, and ecosystem biology have garnered increasing attention. Generally lacking, however, has been consideration of how ubiquitous arbuscular mycorrhizas influence plant allometric and stoichiometric functional traits. We assessed how plant dependence on and responsiveness to mycorrhizas influence plant functional traits of a warm‐season, C4 grass, *Andropogon gerardii* Vitman, and the contrasting, cool‐season, C3 grass, *Elymus canadensis* L. We grew both host species with and without inoculation with mycorrhizal fungi, across a broad gradient of soil phosphorus availabilities. Both host species were facultatively mycotrophic, able to grow without mycorrhizas at high soil phosphorus availability. *A. gerardii* was most dependent upon mycorrhizas and *E. canadensis* was weakly dependent, but highly responsive to mycorrhizas. The high dependence of *A. gerardii* on mycorrhizas resulted in higher tissue P and N concentrations of inoculated than noninoculated plants. When not inoculated, *E. canadensis* was able to take up both P and N in similar amounts to inoculated plants because of its weak dependence on mycorrhizas for nutrient uptake and its pronounced ability to change root‐to‐shoot ratios. Unlike other highly dependent species, *A. gerardii* had a high root‐to‐shoot ratio and was able to suppress colonization by mycorrhizal fungi at high soil fertilities. *E. canadensis*, however, was unable to suppress colonization and had a lower root‐to shoot ratio than *A. gerardii*. The mycorrhiza‐related functional traits of both host species likely influence their performance in nature: both species attained the maximum responsiveness from mycorrhizas at soil phosphorus availabilities similar to those of tallgrass prairies. Dependence upon mycorrhizas affects performance in the absence of mycorrhizas. Responsiveness to mycorrhizal fungi is also a function of the environment and can be influenced by both mycorrhizal fungus species and soil fertility.

## Introduction

Arbuscular mycorrhizal (AM) fungi associate with the vast majority of plant species (Wang and Qiu 2006) and provide their host plants with many benefits, such as pathogen protection (Newsham et al. 1995), improved water relations (Auge 2001), and especially uptake of mineral nutrients such as nitrogen and phosphorus (Smith and Read 2008). Consequently, the degree to which a host species associates with AM fungi may influence its functional traits. Plant functional traits have been defined as measurable morphological, physiological, or phenological properties that affect plant performance (McGill et al. 2006; Westoby and Wright 2006; Friesen et al. 2011). Thus, functional traits can determine where a species establishes, how it interacts with neighboring individuals, and its overall productivity, all of which affect population, community, and ecosystem functioning (McGill et al. 2006). Because AM fungi can increase or decrease plant biomass (Johnson et al. 1997), change root architecture (Hetrick 1991), and increase both nitrogen and phosphorus concentrations within tissues (Smith et al. 2009), they can strongly influence plant functional traits.

Root‐to‐shoot ratio, a well‐known plant functional trait, is a measure of plant belowground versus aboveground carbon allocation. Root‐to‐shoot ratios differ across species and can alter ecosystem carbon dynamics through differences in root metabolism and root turnover (Westoby and Wright 2006). These ratios may evolve in consort with dependence on mycorrhizas such that plant species highly dependent upon mycorrhizas allocate less carbon to root production than those potentially independent of mycorrhizas for nutrient uptake (Hetrick 1991). Phenotypically, mycorrhizal colonization can diminish root‐to‐shoot ratios (Sanders et al. 1977; Veresoglou et al. 2011), potentially in response to elevated plant tissue P concentration (Smith 1980; Ceasar et al. 2014).

Stoichiometric plant functional traits, such as N and P concentrations, may have implications for competition between plant species (Koerselman and Meuleman 1996). Furthermore, plant N:P ratios may indicate potential nutrient limitation within a community, with N:P ratios >16 (Koerselman and Meuleman 1996) or 20 (Güsewell 2004) suggesting P limitation, those <10 (Güsewell 2004) or 14 (Koerselman and Meuleman 1996) suggesting N limitation, and those between 14 and 16 (or 10 and 20) suggesting co‐limitation. Mycorrhizas can increase both N (Hodge and Fitter 2010) and P (Smith and Read 2008) concentrations in plants by adding hyphae to the surface area across which mineral nutrients are absorbed and by hyphae extending beyond zones of nutrient depletion that develop around roots (Smith and Read 2008). Increased mineral nutrient concentrations within plant tissues can affect photosynthesis rate, thereby influencing biomass production (Koerselman and Meuleman 1996; Johnson 2010).

Plant species' reliance on AM fungi for mineral nutrient uptake – their dependence on mycorrhizas – and the change in growth when colonized by mycorrhizas – their responsiveness to mycorrhizas – may explicate mycorrhizas' influence on plant functional traits. The terms “dependence” and “responsiveness” have been used interchangeably to denote a positive effect of mycorrhizas on plant growth (e.g., Hetrick et al. 1986, 1988, 1989; Baon et al. 1993; Wilson and Hartnett 1998; Van Der Heijden and Horton 2009), but Janos (2007) drew a distinction between the terms. Janos (2007) defined mycorrhiza dependence as strictly a property of plant genotype because it is assessed as the inability of a plant species to grow without mycorrhizas. Dependence on mycorrhizas can be measured by the soil P concentration that enables plants without mycorrhizas to reach 10% of their asymptotic growth. The higher the concentration of P required for growth without mycorrhizas, the more “dependent” the plant species. Occasionally, negative concentrations may be calculated for dependence, suggesting that less P than is available in nonfertilized, base soil allows 10% of asymptotic growth.

In contrast to dependence, responsiveness is a property of both plant genotype and the effects of mycorrhizal fungus species on the growth of the plant. Unlike dependence, it is not directly susceptible to natural selection. Responsiveness is measured as the magnitude of plant growth improvement resulting from colonization by mycorrhizal fungi. Responsiveness to mycorrhizas may change with different P fertilities, with high soil fertilities resulting in a small growth difference between inoculated and noninoculated plants. Therefore, maximum responsiveness can be assessed using a gradient of P concentrations to find the largest difference in growth. Additionally, the slopes of such P‐response curves reflect P uptake and use efficiency when a plant is colonized by AM fungi or not, potentially indicating the maximum rate of P conversion to biomass of which a species is capable. Because P‐use efficiency affects plant performance and interactions (Koide 1991), it can be considered a plant functional trait influenced by mycorrhizas.

Mycorrhiza dependence as defined by Janos (2007) determines where plant species lie along the continuum from facultative to obligate mycotrophy, with ecologically obligate mycotrophs incapable of growth at the highest fertility that they naturally encounter. Even though mycorrhizas are beneficial to most plant species in low‐fertility environments, the AM association can range from beneficial, through neutral, to disadvantageous, depending on plant species and environmental conditions (Peng et al. 1993; Johnson et al. 1997; Janos 2007; Smith and Smith 2015). Mycorrhiza disadvantage can result when the carbon cost to the host of sustaining mycorrhizal fungi outweighs the mineral nutrient uptake benefits of mycorrhizas. Such disadvantage often is observed if plants have become heavily mycorrhizal prior to mineral nutrient enrichment (Peng et al. 1993; Janos 2007). Otherwise, in high nutrient environments, facultatively mycotrophic plants can limit the cost of mycorrhizas by suppressing root colonization (e.g., Amijee et al. 1989; Schroeder and Janos 2004; Treseder 2004; Grman 2012).

Grasses are likely to be facultatively mycotrophic thanks to their highly‐branched, extensive fine root systems (Baylis 1972; Janos 1980; Maherali 2014), but C4 grasses, for example, *Andropogon gerardii* Vitman, have been described as “obligately dependent” on mycorrhizas because of their observed inability to grow without mycorrhizas at various single, elevated P availabilities (Hetrick et al. 1986, 1988, 1989; Hartnett et al. 1994; Wilson and Hartnett 1997, 1998). Hetrick et al. (1989) compared the growth of a C3 grass, *Elymus cinereus*, and *A. gerardii*, and found *E. cinereus* growth was depressed by only 46.6% in the absence of AM fungi at a P availability at which *A. gerardii* growth was depressed by 99.5%. Subsequently, Hartnett et al. (1993) found that a lack of mycorrhizas did not significantly affect growth of *Elymus canadensis* L. and that *E. canadensis* was able to outcompete *A. gerardii* when mycorrhizas were absent (but not when they were present). Thus, those authors concluded that C3 grasses generally are less dependent on mycorrhizas than are C4 grasses (Hetrick et al. 1988; Hartnett et al. 1993). Although early research suggested that of *A. gerardii* and *E. canadensis*, only the latter could grow without mycorrhizas (e.g., Hetrick et al. 1986, 1988, 1989), recent studies that repeatedly supplied *A. gerardii* with soluble P have found it too is able to grow without mycorrhizas (Grman 2012; Thorne et al. 2013), hence both species are facultatively mycotrophic (see Janos 2007). Nevertheless, recent work investigating C3 and C4 grasses has concluded that C3 grasses exhibit less of a growth response to mycorrhizas than C4 grasses or exhibit no response at all (Reinhart et al. 2012; Yang et al. 2016). It also is possible that C3, cool‐season grasses are better able to suppress root colonization than highly mycorrhiza‐dependent, C4, warm‐season grasses (Grman 2012), but highly mycorrhiza‐responsive C4 species may show the greatest increases in foliar P content per unit of mycorrhizal root (Treseder 2013).

We sought to assess how allometric and stoichiometric plant functional traits of *A. gerardii* and *E. canadensis* are affected by AM fungi across a gradient of P fertilities. Although many studies have characterized subsets of responsiveness for both species (which those studies called “dependence”), few, if any, studies have characterized dependence as defined by Janos (2007). We sought to assess both dependence and responsiveness, and to determine the level of P at which each species is maximally responsive to mycorrhizas. We hypothesized that: (1) *A. gerardii* would be strongly dependent and *E. canadensis* would be weakly dependent on mycorrhizas for growth, (2) *E. canadensis* would also be weakly responsive to mycorrhizas and have P and N uptake least improved by mycorrhizas (although mycorrhizas would improve the P and N nutrition of both species, and (3) *E. canadensis* would reduce mycorrhizal colonization at high P but *A. gerardii* would not.

## Methods

*Andropogon gerardii* and *Elymus canadensis* are common tallgrass prairies species with contrasting ecologies. *Andropogon gerardii* is a dominant tallgrass prairie species while *E. canadensis* is a subdominant. Often found near *A. gerardii*,* E. canadensis* is usually is found in a mixture of other grasses, constituting about 1–5% of the mixture (Weaver and Fitzpatrick 1934). Because *Andropogon* is genus of tropical origin, its photosynthesis has a high light saturation point and it also has high water‐use efficiency (Turner et al. 1995). In contrast, *E. canadensis* typically is found in lowland areas where it grows best in saturated soils (Weaver and Fitzpatrick 1934). Some ecotypes of *A. gerardii*, however, are tolerant of high soil moisture (Weaver and Fitzpatrick 1934; Olsen et al. 2013).

We grew *A. gerardii* and *E. canadensis* in separate, sequential experiments in an ambient shade house with 30% shade at the Fairchild Tropical Botanic Garden Research Center (Coral Gables, FL). In both experiments, inoculation with AM fungi and weekly soluble phosphorus fertilization treatments was combined fully factorially. Both inoculated and noninoculated *A. gerardii* and *E. canadensis* weekly received 10 mL of seven phosphorus concentrations – 1, 2, 4, 16, 32, 64, 128 *μ*g·g^−1^ P – made with NaH_2_PO_4_ in distilled water, but *E. canadensis* additionally received an 8 *μ*g·g^−1^ P concentration. There were eight individually grown, replicate *A. gerardii* plants and ten *E. canadensis* plants per treatment for totals of 112 *A. gerardii* and 160 *E. canadensis*.

*Andropogon gerardii* and *Elymus canadensis* seeds were obtained from Ever Wilde Farms nursery (Sand Creek, WI) and were germinated on moist paper towels. Approximately 1‐week‐old seedlings were individually transplanted to Ray Leach Cone‐tainers (2.5 cm diameter × 12.1 cm length; 49 mL volume; Tangent, OR). Before transplant, we filled the cone‐tainers with a homogenized mixture of 90% sand and 10% University of Miami Gifford Arboretum soil which was minimally fertile so that we could investigate a broad range of P availabilities. The sand comprised a 3:1 blend of 30–65 grade fine sand and 6–20 grade coarse sand from Florida Silica Sand Company (Miami, FL). The soil mixture was autoclaved at 121°C and 1.4 kg·cm^−2^ for 1 h, three times, each 24 h apart, and was used for all treatments of both experiments. The pasteurized soil mixture had a pH of 7.7, 3.2 *μ*g·g^−1^ ammonium N, 2.6 *μ*g·g^−1^ nitrate N, 12.5 *μ*g·g^−1^ Olsen P, 48.8 *μ*g·g^−1^ K, 355.8 *μ*g·g^−1^ Ca, 48.3 *μ*g·g^−1^ Mg, and 1.1 *μ*g·g^−1^ Mn according to analyses by the Kansas State Agronomy Soil Testing Laboratory (Manhattan, KS).

We inoculated cone‐tainers with multiple species of mycorrhizal fungi contained in pieces of fine roots of *Stenotaphrum secundatum* (Walt.) Kuntze lawn grass collected from the Gifford Arboretum. The roots contained *Sclerocystis rubiformis* Gerd. & Trappe, *Glomus clarum* Nicholson & Schenck (now *Rhizophagus clarus* Walker & Schußler) and several species of *Glomus sensu lato* (Weremijewicz and Janos 2013). Freshly collected roots were cut into 1–2 cm pieces by hand, and 110 g of roots was mixed uniformly throughout the entire soil volume before the cone‐tainers were filled. Noninoculated cone‐tainers received the same weight of root pieces that had been autoclaved for 1 h, three times, 24 h apart to partially control for the organic matter addition to inoculated plants. Noninoculated plants also received a microbial filtrate made by soaking 110 g of freshly collected *S. secundatum* roots in 1 L of water for 24 h and then filtering the solution through Whatman #4 filter paper to exclude AM fungi. The microbial filtrate was added to the soil of the noninoculated treatment before filling cone‐tainers. Soil of the inoculated cone‐tainers was given the same volume of distilled water. For *E. canadensis*, we additionally inoculated each cone‐tainer with 1 mL (*ca*. 333 spores/mL) of a commercial inoculant (Mycorrhizal Applications, Grants Pass, OR) to try to avoid any inoculum limitation. The slurry of spores and root fragments was pipetted into the transplant hole. It contained four “*Glomus”* species (*G. intraradices* Schenck & Smith [now *Rhizophagus intraradices* (Krüger et al. 2012)]*, G. etunicatum* Becker & Gerd. [now *Claroideoglomus etunicatum* Walker & Schußler]*, G. mosseae* Gerd. & Trappe [*now Funneliformis mosseae* Walker & Schußler], and *G. aggregatum* Schenck & Smith). At the same time, we gave noninoculated plants an equal amount of spore slurry autoclaved as described above. Because of the potentially different suites of fungi that could have colonized *A. gerardii* and *E. canadensis*, and the different years in which we conducted their experiments, we focus on within‐species allometry and stoichiometry rather than directly comparing the plant species' performance.

*Andropogon gerardii* and *Elymus canadensis* were grown for 77 and 71 days after transplant (DAT), respectively, until growth began to slow. *Andropogon gerardii* was grown from August 7, 2013 to October 22, 2013 (average daily temperature = 28.1 ± 1.2°C SD) and *E. canadensis* from August 1, 2014 to October 10, 2014 (average temperature = 27.0 ± 1.2°C SD). Beginning 7 DAT, we fertilized all cone‐tainers twice per week: once with 10 mL of the designated P treatment and 2 days later with 10 mL of Hewitt's solution (lacking phosphate) with concentrations of: 2 mmol·L^−1^ KNO_3_, 5 mmol·L^−1^ Ca(NO_3_)_2_, 1.5 mmol·L^−1^ MgSO_4_·7H_2_O, 0.1 mmol·L^−1^ Ferric Citrate, 0.03 mmol·L^−1^ H_3_BO_3_, 0.011 mmol·L^−1^ MnSO_4_, 0.002 mmol·L^−1^ ZnSO_4_, 0.0003 mmol·L^−1^ (NH_4_)_6_Mo_7_O_24_·4H_2_0, and 0.1 mmol·L^−1^ CuSO_4_·5H_2_0.

Every week, we measured the length of the longest leaf of each plant to determine when growth began to slow. At harvest, we clipped shoots directly above the basal meristem and dried them for 7 days to constant weight at 60°C for determination of shoot dry weight. We extracted root systems in gently running water over a 1 mm sieve and blotted them dry prior to determining their fresh weight. Subsequently, after randomly removing a subsample of fine roots from each root system and re‐weighing the remaining roots, the remaining roots were dried to constant weight at 60°C. We used the dry weight to fresh weight ratio to calculate the dry weights of entire root systems. Subsampled roots were preserved in 50% ethanol until being stained and examined for AM fungus colonization. Shoot and root dry weight data were summed to calculate the total dry weight of each plant. Dried leaf tissues were composited by treatment, and foliar N and P concentrations were determined by the Kansas State Agronomy Soil Testing Laboratory (Manhattan, KS).

We assessed the percentage root length colonized by AM fungi for the preserved, subsampled roots. We cleared the roots in 10% KOH for 3 days, acidified them in 5% HCl for 30 min, and then placed them in 0.05% Trypan blue in lactoglycerol for 24 h, all at room temperature. For *A. gerardii*, we scored mycorrhizal colonization from six randomly selected plants per treatment. We placed 35 one‐centimeter root segments from each plant on microscope slides and examined 105 magnified gridline intersections per plant (McGonigle et al. 1990). For *E. canadensis*, we combined root segments from all plants within a treatment, mixed them well, and mounted 67 segments from each treatment on slides, examining 201 intersections per treatment. We distinguished AM fungi at gridline intersections by the presence of blue‐stained coenocytic external and internal hyphae with occasional unilateral projections, vesicles, or arbuscules.

### Statistical analyses

We fitted phosphorus response curves to shoot and total dry weight data separately for *A. gerardii* and *E. canadensis* with Statistix v. 10.0 (Analytical Software, Tallahassee, FL) to minimize squared deviations. The fitted curves conformed to the logistic equation given by Janos (2007), *W *= *A*/(1 + *b* · exp(−*SP*)), where *b *= (*A* − *I*)/*I, W* represents shoot dry weight, *P* is the concentration of added phosphorus, *I* is the *y*‐axis intercept, *A* is the asymptote, and *S* is the slope of a tangent at the inflection point. We assessed normality of the residuals around the nonlinear regression fitted curve using the Shapiro–Wilk normality test for each line. When the Shapiro–Wilk test indicated non‐normal residuals, we rank ordered the residuals, and eliminated them one point at a time until the data fit the normality assumption. This resulted in the elimination of four points from the aboveground DW curve (one point from the 1 *μ*g·g^−1^, 16 *μ*g·g^−1^, 64 *μ*g·g^−1^ and 128 *μ*g·g^−1^ P treatments) and two points from the total DW curve (one point from 64 *μ*g·g^−1^ and 16 *μ*g·g^−1^ P treatments) for the inoculated *A. gerardii* treatment.

Maximum responsiveness (denominated in grams) was calculated from the fitted curves as the largest vertical, positive difference for inoculated minus noninoculated plants (Janos 2007). Dependence upon mycorrhizas (denominated in units of phosphorus concentration) was calculated as the phosphorus concentration at which plants without mycorrhizas attained 10% of their asymptotic size under our experimental conditions.

We examined each host species' growth responses and foliar N and P concentrations with two‐way, factorial analysis of variance (ANOVA). One *A. gerardii* individual with a root dry weight five standard deviations above the mean of the other plants in its treatment, and one *E. canadensis* with a total dry weight eight standard deviations below the mean of the other plants in its treatment were omitted from all analyses. Only, total dry weights were heteroscedastic, and so were log‐transformed. We calculated root‐to‐shoot ratios by dividing root dry weights by shoot dry weights, and then, we examined the mean ratios for inoculated and noninoculated plants using least‐squares linear regressions versus log‐transformed P concentrations. All statistical testing was performed with Statistix v. 10.0 using *ɑ *= 0.05 to determine significance.

We visualized foliar N and P concentrations and contents with vector graphs (Swift and Brockley 1994; Haase and Rose 1995; Scagel 2003). For each plant species, we relativized N and P concentrations and total dry weight versus the 1 *μ*g·g^−1^ treatment of noninoculated and inoculated treatments separately.

We examined effects of treatments on root colonization by AM fungi with two‐way, factorial ANOVAs for each host species separately, using P amendment and inoculation as factors. After testing percent colonized root length for heteroscedasticity with Levene's test, we arcsine‐square root transformed all colonization data. We examined potential associations between nontransformed percentage root length colonized by AM fungi and log‐transformed P concentrations (to reduce curvilinearity) by calculating Pearson's correlation coefficients. Association analyses of mycorrhizal colonization involved only inoculated plants.

## Results

### Plant growth and allometry

Plants in all treatments began the experiment similarly sized. At the first measurement at 7 DAT prior to beginning fertilization, mean longest leaf lengths of *A. gerardii* (two‐way ANOVA, MYC: *F*_1,98_ = 3.18, *P *=* *0.08, P concentration: *F*_6,98_ = 0.39, *P *=* *0.8821, MYC × P concentration: *F*_6,98_ = 0.17, *P *=* *0.9834) and of *E. canadensis* (two‐way ANOVA, MYC: *F*_1,144_ = 0.46, *P *=* *0.4999, P concentration: *F*_7,144_ = 1.18, *P *=* *0.3184, MYC × P concentration: *F*_6,98_ = 1.02, *P *=* *0.4216) were not statistically distinguishable.

**Figure 1:**
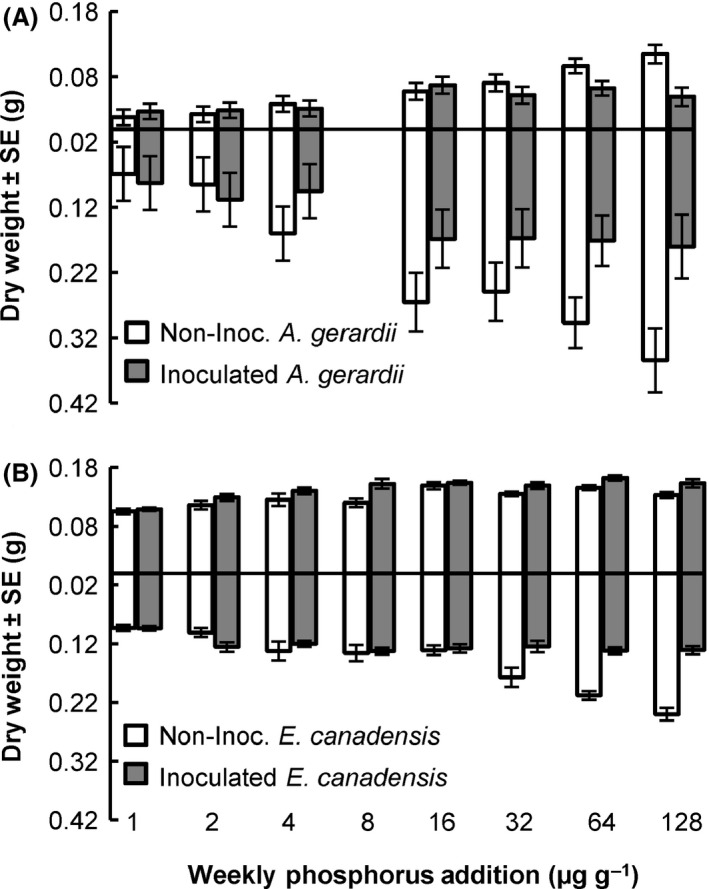
Mean dry weights ± SE (g) aboveground (bars above the *x*‐axis) and belowground (positive‐value bars below the *x*‐axis) of noninoculated plants or inoculated plants versus the concentrations of 10 mL weekly phosphorus additions (*μ*g·g^−1^) for *Andropogon gerardii* (A) and *Elymus canadensis* (B). ANOVA results are shown in Table 1.

At harvest, the main effect of P addition and the interaction between inoculation and P addition were significant for shoot, root, and total dry weight of *A. gerardii*, and the main effect of inoculation was significant only for root and shoot dry weights (Table 1). Both shoot and root dry weights of *A. gerardii* increased with increasing P fertilization (Fig. 1A). Phosphorus addition had a significant main effect on *E. canadensis* shoot, root, and total dry weights (Table 1). Shoot and root dry weights showed a significant effect of inoculation, but total dry weights did not (Table 1). Root dry weights and total dry weights also showed a significant interaction between P addition and inoculation (Table 1). Inoculated *E. canadensis* plants were consistently heavier in shoot weights than noninoculated plants, and shoot weights of both noninoculated and inoculated plants increased with increasing concentrations of P fertilizer (Fig. 1B). Root dry weights of noninoculated plants remained approximately the same at P additions 16 *μ*g·g^−1^ or less but exceeded inoculated plants at P additions of 32 *μ*g·g^−1^ or greater, thereby increasing total dry weights of noninoculated plants at these P additions (Fig. 1B).

**Table 1:** Two‐way, factorial ANOVA (MYC = presence/absence of mycorrhizas and P = concentration of weekly phosphorus addition) results for shoot and root dry weights and total dry weights at harvest 76 days after transplant (DAT) for *Andropogon gerardii* and 70 DAT for *Elymus canadensis*

Growth parameter	Factor	*Andropogon gerardii*			*Elymus canadensis*		
		df	*F*	*P*	df	*F*	*P*
Shoot Dry Weight	MYC	1,70	2.64	0.1084	1,143	**21.51** a	**<0.0001**
	P	6,70	**6.92**	**<0.0001**	7,143	**11.67**	**<0.0001**
	MYC x P	6,70	**2.84**	**0.0156**	7,143	0.98	0.4505
Root Dry Weight	MYC	1,70	**9.77**	**0.0026**	1,143	**24.58**	**<0.0001**
	P	6,70	**5.15**	**0.002**	7,143	**13.28**	**<0.0001**
	MYC x P	6,70	**2.68**	**0.0211**	7,143	**7.70**	**<0.0001**
Total Dry Weight	MYC	1,70	**8.19**	**0.0056**	1,143	3.17	0.0771
	P	6,70	**5.85**	**0.0001**	7,143	**15.17**	**<0.0001**
	MYC x P	6,70	**2.81**	**0.0166**	7,143	**4.01**	**0.0005**

a Significant differences (*α *= 0.05) are shown in bold.

**Figure 2:**
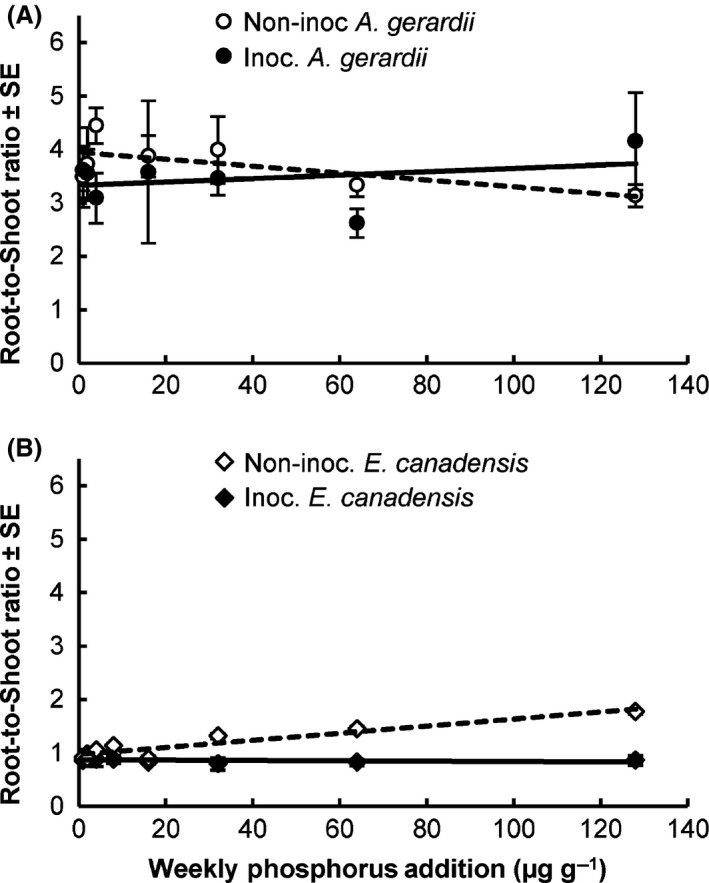
Mean root‐to‐shoot ratios (root dry weight/shoot dry weight ± SE) versus concentrations of 10 mL weekly phosphorus additions (*μ*g·g^−1^) for inoculated (solid lines) and noninoculated (dashed lines) *Andropogon gerardii* (A) and *Elymus canadensis* (B) plants.

Root‐to‐shoot ratios decreased with increasing P fertilization for noninoculated *A. gerardii* (*F*_1,38_ = 10.78, *P *=* *0.022; R/S = −0.01*P concentration + 4.35; Fig 2A) but were not affected significantly for inoculated plants of either *A. gerardii* (*F*_1,42_ = 0.09, *P *=* *0.7693; Fig. 2A) or *E. canadensis* (*F*_1,77_ = 0.87, *P *=* *0.3544; Fig. 2B). Noninoculated *E. canadensis*, however, showed a significant increase in root‐to‐shoot ratio as P fertilizer concentration increased (*F*_1,78_ = 62.81, *P *<* *0.0001; R/S = 0.006*P concentration + 0.97; Fig. 2B). Although we did not compare them statistically, it is important to note that the grand mean root‐to‐shoot ratio of *A. gerardii* was 3–4 times the mean ratio for inoculated *E. canadensis* (Fig. 2).

**Figure 3:**
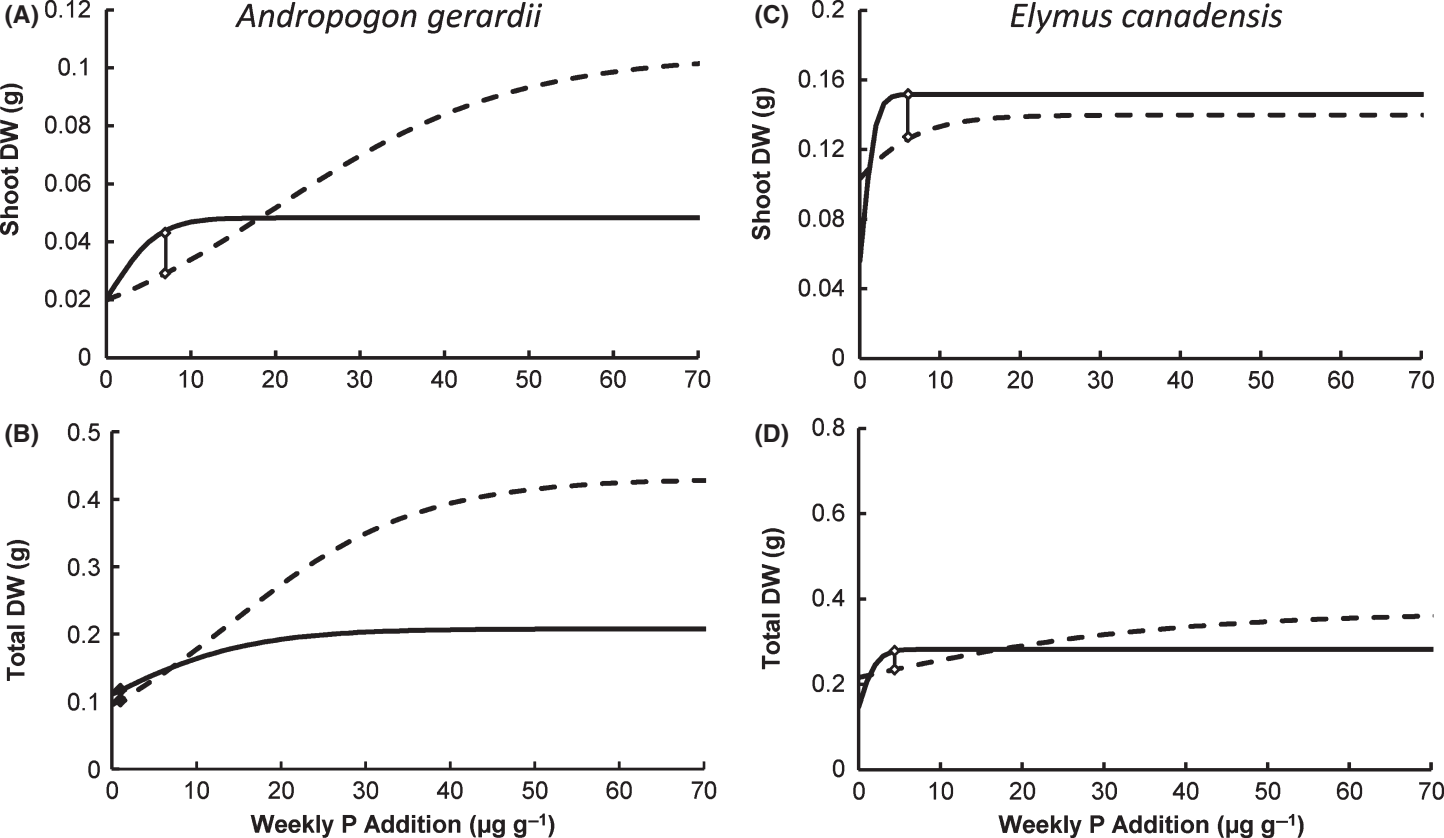
Logistic phosphorus response curves fitted to shoot dry weights (DW, g; A, C) and total dry weights (B, D) versus concentrations of 10 mL weekly phosphorus additions (*μ*g·g^−1^) for *Andropogon gerardii* (A, B) and *Elymus canadensis* (C, D) noninoculated (dashed lines) and inoculated (solid lines) with arbuscular mycorrhizal fungi. Absolute maximum responsiveness to mycorrhizas is represented by solid vertical lines connecting open diamonds in each panel. Parameters for each curve are shown in Table 2.

We fitted logistic curves to both total dry weights and shoot dry weights to compare their interpretations, particularly because root‐to‐shoot ratios differed among inoculation and P addition treatments. When we fitted curves for shoot dry weights of noninoculated plants, we calculated that the dependence of *A. gerardii* was at −5.0 *μ*g·g^−1^ P. In other words, 10% of its asymptotic growth was predicted to occur at an estimated 5.0 *μ*g·g^−1^ P less than was available in the base substrate. Mycorrhiza disadvantage occurred above 18.1 *μ*g·g^−1^ P and *A. gerardii* had maximum responsiveness to mycorrhizas at 7.0 *μ*g·g^−1^ P, with the shoots of inoculated plants 1.5 times heavier than those of noninoculated plants (Table 2; Fig. 3A). Curves fitted to total dry weights revealed that mycorrhiza dependence was estimated to be −19.7 *μ*g·g^−1^ P, mycorrhizas were disadvantageous above 7.1 *μ*g·g^−1^ P (Fig. 3B), and inoculated *A. gerardii* plants were 1.1 times heavier than noninoculated plants at 1.0 *μ*g·g^−1^ P (Table 2). We calculated *E. canadensis*'s mycorrhiza dependence to be −16.6 *μ*g·g^−1^ P (Table 2) and a maximum responsiveness to mycorrhizas of 1.1 times the shoot weight of noninoculated plants at 7.0 *μ*g·g^−1^ P (Table 2; Fig. 3C). Although mycorrhizas were not disadvantageous for *E. canadensis* based upon shoot dry weights (Table 2; Fig. 3C), total plant dry weights revealed that mycorrhizas were disadvantageous above 20.8 *μ*g·g^−1^ P (Fig. 3D). *Elymus canadensis* total dry weight mycorrhiza dependence was estimated as −52.6 *μ*g·g^−1^ P (Table 2), maximum responsiveness at 4.4 *μ*g·g^−1^ P at which inoculated plants were 1.2 times heavier than noninoculated plants.

**Table 2:** Parameters, dependence, and responsiveness from logistic phosphorus response curves fitted to shoot dry weights (DW) and total dry weights versus concentrations of weekly phosphorus additions for noninoculated (Noninoc.) and inoculated (Inoc.) *Andropogon gerardii* and *Elymus canadensis* plants

Parameter	*Andropogon gerardii*				*Elymus canadensis*			
	Shoot DW		Total DW		Shoot DW		Total DW	
	Noninoc.	Inoc.	Noninoc.	Inoc.	Noninoc.	Inoc.	Noninoc.	Inoc.
Intercept (g)	0.02	0.02	0.09	0.11	0.10	0.06	0.22	0.15
Slope (g DW/P *μ*g·g^−1^)	0.07	0.37	0.09	0.12	0.20	1.3	0.05	0.91
Asymptote (g)	0.10	0.05	0.43	0.21	0.14	0.15	0.37	0.28
Dependence (P *μ*g·g^−1^)	−5.0		−19.7		−16.6		−52.6	
Maximum responsiveness (g)	0.015		0.015		0.015		0.043	
P concentration for maximum responsiveness (*μ*g·g^−1^)	7.0		1.0		7.0		4.4	
Upper P concentration (*μ*g·g^−1^) at which noninoculated and inoculated P‐response curves intersect	18.1		7.1		NA		20.8	

NA, not available.

### Phosphorus and nitrogen stoichiometry

Mycorrhizas improved P and N nutrition for *A. gerardii* but not for *E. canadensis*. For all P additions, noninoculated *A. gerardii* individuals had lower mean foliar P (*F*_1,6_ *= *9.69, *P *=* *0.0208) and N concentrations (*F*_1,6_ *= *7.91, *P *=* *0.0307) than inoculated individuals (Table S1). Noninoculated and inoculated *E. canadensis* foliar mean concentrations did not differ for either P (*F*_1,7_ = 0.64, *P *=* *0.4508) or N (*F*_1,7_ *= *2.68, *P *=* *0.1348; Table S1).

**Figure 4:**
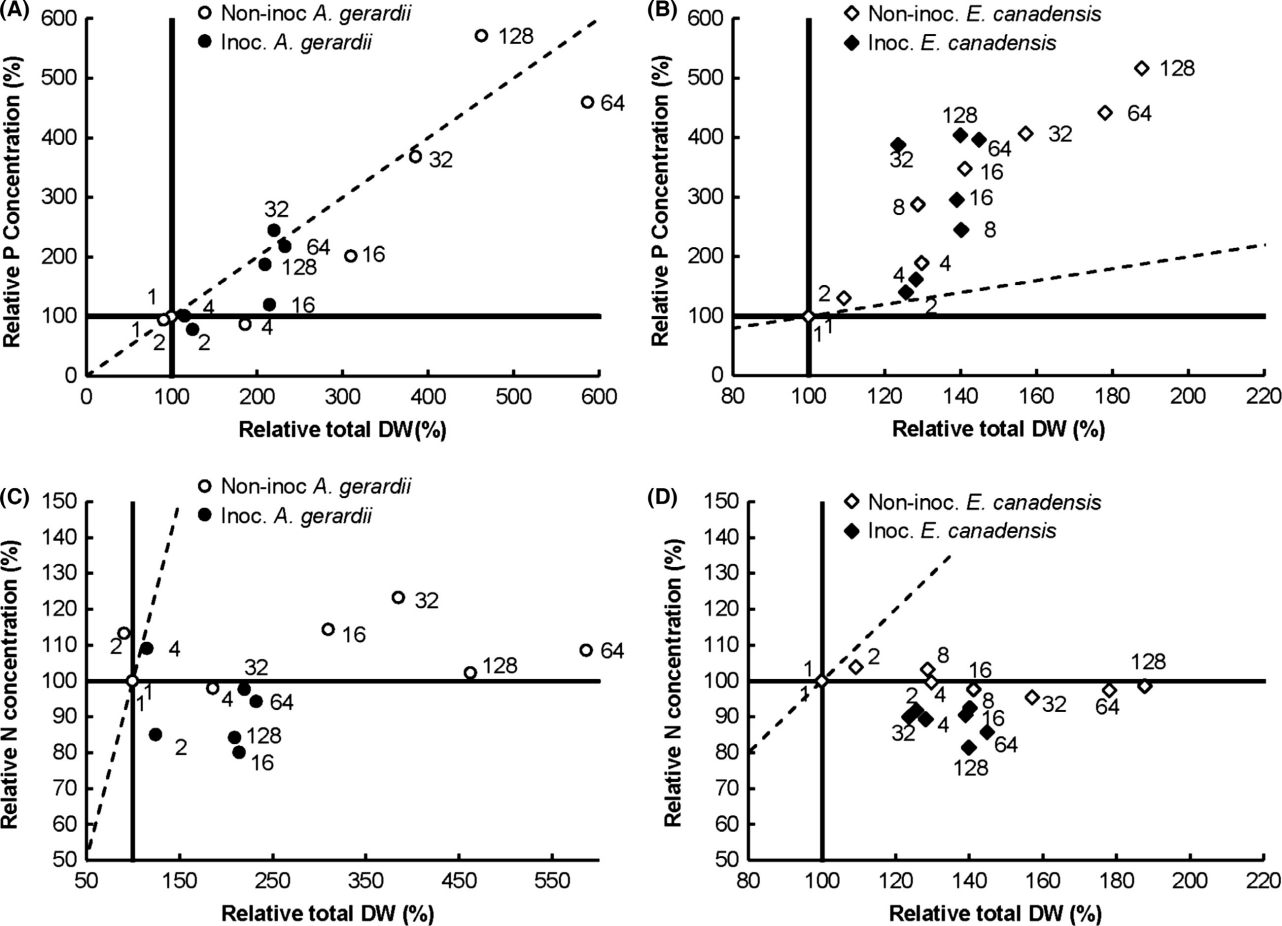
Comparison of leaf tissue relative phosphorus (A, B; 100 * [P_conc_]/[P_1 *μ*g·g_^−1^] where P_conc_ represents each P addition concentration) and nitrogen (C, D; calculated similarly to relative P) concentrations versus relative total dry weight (DW; calculated similarly to relative P concentrations) for *Andropogon gerardii* (A, C) and *Elymus canadensis* (B, D) noninoculated or inoculated with arbuscular mycorrhizal fungi at different concentrations of weekly 10 mL P additions (*μ*g·g^−1^ noted next to symbols). The vertical and horizontal heavy solid lines are at 100% DW and P or N, respectively. The dashed diagonal line represents equal proportional changes in leaf tissue relative phosphorus concentration and relative total dry weight; points above the diagonal line, between it and the heavy vertical line, suggest luxury accumulation and storage, while points between the diagonal line and the heavy horizontal line suggest dilution by plant growth (see Scagel 2003).

Vector graphs of relative mean foliar P concentrations per treatment versus relative mean total dry weights (relativized by the 1 *μ*g·g^−1^ P treatment) showed that at and above P fertilizer concentrations of 32 *μ*g·g^−1^ P, both noninoculated and inoculated *A. gerardii* tended to concentrate P in proportion to their weight (Fig. 4A). For inoculated *A. gerardii*, however, mean relative dry weight changed little at and above 16 *μ*g·g^−1^ P addition, but the mean relative dry weights of noninoculated plants tended to increase with increasing P fertilizer concentration. Both noninoculated and inoculated *E. canadensis* tended to accumulate luxury P (Fig. 4B), but similar to inoculated *A. gerardii*, the relative mean dry weights of inoculated *E. canadensis* changed little with increasing P fertilizer concentration while the relative mean total dry weights and foliar P concentrations of noninoculated plants both increased.

Nitrogen vector graphs for both inoculated *A. gerardii* (Fig. 4C) and inoculated *E. canadensis* (Fig. 4D) suggest a slight dilution of foliar N with increased P fertilizer concentration, although there was little change in relative mean dry weight for either species at 8 *μ*g·g^−1^ added P or higher. Noninoculated plants of both species, however, tended to maintain little‐changed relative mean concentrations of foliar N even though relative mean total dry weights increased with increasing P fertilizer concentration.

**Figure 5:**
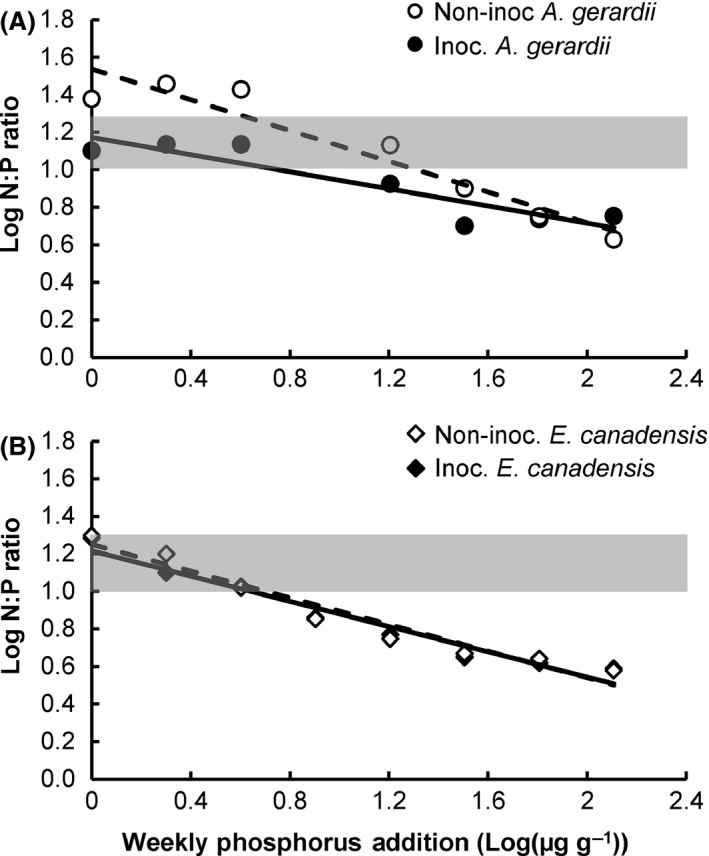
Log‐transformed N:P ratios (Log_10_([N]/[P]) versus log‐transformed concentrations of weekly P additions (Log_10_[*μ*g·g^−1^]) for inoculated (solid lines) and noninoculated (dashed lines) *Andropogon gerardii* (A) and *Elymus canadensis* (B). The shaded area indicates N:P between 10 and 20 (Güsewell 2004) which separates potential N limitation (above the shading) from potential P limitation (below the shading). N and P are potentially co‐limiting within the shaded area.

N:P ratios decreased with increasing P fertilizer concentrations for inoculated (*F*_1,6_ = 26.24, *P *=* *0.0037; N:P = −0.23*P concentration + 1.17) and noninoculated (*F*_1,6_ = 54.35, *P *=* *0.0007; N:P = −0.41*P concentration + 1.53) *A. gerardii* plants (Fig. 5A; Table S1), but at different rates (*F*_1,10_ = 6.54, *P *=* *0.0285). N:P ratios decreased at similar rates (*F*_1,12_ = 0.18, *P *=* *0.6814) with increasing P fertilizer concentrations for both inoculated (*F*_1,7_ = 131.63, *P *<* *0.00001; N:P = −0.33*P concentration + 1.21) and noninoculated (*F*_1,7_ = 108.04, *P *< 0.00001; N:P = −0.36*P concentration + 1.25) *E. canadensis* plants (Fig. 5B). At low P fertilizer concentrations below 8 *μ*g·g^−1^, noninoculated *A. gerardii* exhibited N:P ratios greater than 20, suggesting potential P limitation of growth, but inoculated *A. gerardii* and all *E. canadensis* may have been co‐limited by P and N. At and above 8 *μ*g·g^−1^ P (except for noninoculated *A. gerardii* at 16 *μ*g·g^−1^ P) all plants of both species had N:P ratios consistent with potential N limitation.

### Root colonization

Root colonization by AM fungi was negatively correlated with weekly P addition (*n* = 42, Pearson's *r *=* *−0.47, *P *=* *0.0084) only for *A. gerardii*, and not significantly so for *E. canadensis* (*n* = 8, Pearson's *r *=* *−0.63, *P *=* *0.0960). *A. gerardii* had the greatest colonization by AM fungi at 4 *μ*g·g^−1^ P with an average of 51% root length colonized, and *E. canadensis* had the greatest colonization at 1 *μ*g·g^−1^ P with 19% root length colonized despite having received the commercial inoculum in addition to locally collected inoculum. Inoculated *A. gerardii* individuals fertilized with 64 *μ*g·g^−1^ P had 6% colonization, their lowest, but *E. canadensis* had their lowest colonization, 10%, at 4 *μ*g·g^−1^ P. Noninoculated plants of both species mostly remained without mycorrhizas for the duration of the experiments. Only four *A. gerardii* among 42 noninoculated plants had any colonization: three of those had less than 1.5% colonization and the forth had 5% colonization. Noninoculated *E. canadensis* (composited by P fertilizer concentration) had 0.4% colonization at only a single P fertilizer concentration. Because colonization was minimal, we retained these plants in analyses.

## Discussion

### Plant growth and allometry

*Andropogon gerardii* and *Elymus canadensis* were facultatively dependent on mycorrhizas for growth. Although *A. gerardii* was strongly dependent on mycorrhizas as we hypothesized, it was not literally an “obligate” mycotroph as previously suggested (Hetrick et al. 1986, 1988, 1990; Hartnett et al. 1993). In our study, noninoculated *A. gerardii* were able to equal the growth of inoculated plants with as little as an estimated 18.1 *μ*g·g^−1^ P supplied weekly (totaling 28 mg·L^−1^ soil over our entire experiment), making it a facultative mycotroph as found recently by others (Miller et al. 2002; Grman 2012; Thorne et al. 2013). When the total amount of P applied throughout an experiment is summed, our results are similar to those of Grman (2012) who found mycorrhiza disadvantage for *A. gerardii* at total P additions above 86 mg·L^−1^ soil and Thorne et al. (2013) who found disadvantage above 49 mg·L^−1^ soil total P addition. Thorne et al. (2013) used field collected soil, but Grman (2012) used a nutrient‐poor sand‐soil mixture like ours, so base substrate differences in P availability undoubtedly contribute to the somewhat different threshold values for mycorrhiza disadvantage. Although Hetrick et al. (1986) had found noninoculated *A. gerardii* did not respond to 30 *μ*g·g^−1^ P with improved growth, they added dry, soluble P only once at the start of their experiment, providing a total P addition of only 0.01 mg·L^−1^ soil. Hence, we suggest that *A. gerardii* is physiologically facultatively mycotrophic when substrate P concentrations are maintained artificially high, but it may be “ecologically obligately mycotrophic” (Janos 1980), incapable of survival and growth without mycorrhizas at naturally occurring concentrations of P, which generally are 5–20 *μ*g·g^−1^ in tallgrass prairies (Bray 1; Johnson et al. 2010).

The least dependent species, *E. canadensis,* was not the least responsive as hypothesized, which underscores the merits of distinguishing between “dependence” and “responsiveness.” Both *A. gerardii* and *E. canadensis* achieved maximum responsiveness, at similar P fertilizer concentrations of 1.0–7.0 *μ*g·g^−1^, likely within the natural range of P concentrations in prairies, because our base substrate contained 12.5 *μ*g·g^−1^ Olsen P and our soluble P additions effectively were pulsed because of leaching by subsequent watering. We found that *E. canadensis* can greatly increase carbon allocation belowground, thereby potentially compensating for a lack of mycorrhizas, which is consistent with it being less dependent on mycorrhizas than *A. gerardii*. Aboveground data alone, however, failed to indicate that mycorrhizas could be disadvantageous to *E. canadensis*, and they additionally gave a dependence estimate only one‐third that calculated from total dry weight. That provides a caveat for interspecific competition studies in which the roots of different species are difficult to disentangle such that only aboveground dry weight data are considered.

Plant species with high root‐to‐shoot ratios have been thought to be less responsive to mycorrhizas than those with low ratios (Koide et al. 1988), but a recent meta‐analysis by (Maherali 2014) found no relationship between root traits and responsiveness to mycorrhizas (sensu Janos 2007). In our study, although we found *A. gerardii* to be the most dependent on mycorrhizas, it had a high root‐to‐shoot ratio that was three times greater than that of *E. canadensis*. We were not able to measure specific root length, but we observed that the roots of *A. gerardii* are considerably coarser than those of *E. canadensis*, and this probably explains the high root‐to‐shoot biomass ratio of *A. gerardii*. High specific root lengths, as we infer for *E. canadensis*, may characterize at least some plant species well able to take up mineral nutrients without mycorrhizas (Hetrick 1991).

Greater proportional allocation to roots by noninoculated *E. canadensis* than by *A. gerardii* is consistent with the different abilities of the species to grow without mycorrhizas. Noninoculated *E. canadensis* increased root production and *A. gerardii* decreased root production as P fertilizer concentration increased, but inoculated plants did not change root‐to‐shoot ratios. Increased biomass allocation to roots is a common response to N and P deficiencies (Güsewell 2004), although it is especially strongly associated with N limitation (Andrews et al. 1999). If *E. canadensis* experiences a flush of mineral nutrient release from accelerated organic matter decomposition in the early spring when it typically begins to grow (Weaver and Fitzpatrick 1934), and if mycorrhizal fungus activity is retarded by low night temperatures (Liu et al. 2004) or saturated soils, then *E. canadensis* might be under strong natural selection to prioritize root production.

### Phosphorus and nitrogen stoichiometry

Plant tissue P and N concentrations are acknowledged plant functional traits (McGill et al. 2006; Westoby and Wright 2006; Friesen et al. 2011), and our work illustrates how arbuscular mycorrhizas can affect them. When plants were not inoculated, as soil P fertilizer concentration increased, relative tissue P concentrations and relative total dry weights tended to increase similarly for *A. gerardii*, but tissue P increased much more rapidly than dry weight for *E. canadensis*, perhaps because of its increasing root‐to‐shoot ratio. When inoculated, however, both *A. gerardii* and *E. canadensis* accumulated luxury P at and above 8 *μ*g·g^−1^ weekly P fertilization, consistent with something other than P limiting growth.

Inoculation increased P‐response curve slopes, which can be used to quantify P uptake and use efficiency (Koide 1991) because they reflect the maximum rate of uptake and conversion of a unit increase in P availability into plant biomass. Surprisingly, both plant species without mycorrhizas had similar P uptake and use efficiencies based upon total dry weight. As the less mycorrhiza‐dependent species, we expected *E. canadensis* to have an ability to acquire and use P efficiently without mycorrhizas. Similar slopes for both *E. canadensis* and *A. gerardii* without mycorrhizas suggest similar physiologies between the two plant species, and so the differences between them when mycorrhizal are most likely because of differences in P acquisition.

In accord with our hypothesis, mycorrhizas increased the foliar concentrations of both P and N in *A. gerardii*. Despite our use of a nutrient‐poor sand mixture, both *A. gerardii* and *E. canadensis* had foliar P and N concentrations similar to or in excess of previously reported values for plants in native soils, suggesting that our plants were not abnormal. Kemp et al. (1995), Loaiza et al. (2011), and Griffin and Jung (1983) reported *A. gerardii* P concentrations of 0.09–0.2%, and most of our values fall within or above that range except for noninoculated plants at the three lowest P fertilizer concentrations. Our values also mostly fall within the 1.0–1.5% range of N concentrations reported for *A. gerardii* by Delucia et al. (1992), Loaiza et al. (2011), and Owensby et al. (1993). Our *E. canadensis* grown at P fertilizer concentrations above 8 *μ*g·g^−1^ had P tissue concentrations higher than the 0.09–0.17% reported for plants in native soils (Klabi et al. 2014), while N concentrations at all P fertilizer concentrations exceeded the 0.83–1.43% reported by Klabi et al. (2014). It is puzzling how *E. canadensis* was the most responsive of our two host species when neither its foliar P nor N concentrations differed between noninoculated and inoculated plants across all P fertilizer concentrations. Perhaps inoculated *E. canadensis* tissue P concentrations were elevated in roots instead of in shoots, or another element other than P or N limited growth and mycorrhizas increased its uptake.

Generally low and decreasing N:P ratios suggest that N limitation increased for both plant species as P fertilizer concentration was increased. Vector analyses, however, show relative N concentrations remained essentially constant with increasing relative dry weights of both plant species when not inoculated and generally diminished with increased relative dry weights of inoculated plants. Increased P concentrations at high soil P fertilizer additions and constant N uptake likely led to decreasing N:P ratios for both plant species. As the less dependent plant, *E. canadensis* was able to increase its root‐to‐shoot ratio to capture N at high P fertilizer, which may have resulted in N:P ratios similar to those of inoculated plants. For *A. gerardii*, the presence of mycorrhizas may have increased N uptake, as suggested by higher N:P ratios for inoculated plants than for noninoculated plants. N:P ratios reflect the balance between N, P, and C within plant tissues (Güsewell 2004), and at high P fertilizer concentrations, it is likely that the carbon cost of mycorrhizas limited the growth of both host species. Even though increasing P fertilization did diminish percentage colonized root length, neither host species fully suppressed mycorrhizas, perhaps because our soluble P additions were pulsed.

### Root colonization

Facultatively mycotrophic plant species are thought to suppress colonization and to decrease carbon allocation to AM fungi to reduce the cost of nonbeneficial mycorrhizas (Treseder 2004; Kiers et al. 2011). Nevertheless, positive growth responses to mycorrhizas by both host species in our experiments suggests that the AM fungus species we used were effective mutualists at least at low soil P availability.

As we increased phosphorus fertilizer concentration, however, percent colonized root length did decrease significantly for *A. gerardii*. The high Pearson's correlation coefficient of −0.63 for *E. *canadensis suggests that its colonization also diminished with increased P fertilization and that its lack of significance most likely was a consequence of a small sample size. Colonization of *E. canadensis* in our study was similar to that reported by Wilson and Hartnett (1998), around 15%.

The significant negative relationship (Pearson's *r *=* *−0.47) between P fertilization and root colonization for *A. gerardii* appears to contradict Grman's (2012) finding that highly mycorrhiza‐dependent, C4 plants are unable to suppress mycorrhizal colonization. Abundant root production by *A. gerardii* at P fertilizer concentrations of 16 *μ*g·g^−1^ and higher, however, effectively may have “diluted” colonization by AM fungi that may not have spread as quickly as roots grew. The greatest mean root colonization of both *A. gerardii* and *E. canadensis* coincided with their lowest mean root dry weights, but because we did not measure fine root length, we do not know if the total length of mycorrhizal roots remained constant or diminished as P fertilizer concentration was increased. Regardless of possible reductions of mycorrhizal root length, both host species when inoculated were mycorrhizal at all P fertilizer concentrations.

Both host species received similar wild‐collected inoculum, but the four additional *Glomus* species provided to *E. canadensis* might have enhanced its P acquisition, thereby making its responsiveness to mycorrhizas similar to that of *A. gerardii*. Different species of AM fungi can differentially benefit hosts (Kiers et al. 2011). Nevertheless, total root colonization was low for *E. canadensis*, consistent with it being less dependent upon mycorrhizas than *A. gerardii*.

## Conclusion

Plant functional traits that are influenced by mycorrhizas – P uptake and use efficiency, root‐to‐shoot ratios, and foliar P and N concentrations – likely strongly influence the ecological niches of plant species. In particular, requirement of mycorrhizas, “mycorrhiza dependence,” can restrict establishment of a species in sites lacking AM fungal inocula, such as postagricultural fields (Kurle and Pfleger 1994; Richter and Stutz 2002) and mine reclamation sites (Gould and Liberta 1981; Thorne et al. 2013). We have shown that both *A. gerardii* and *E. canadensis* are able to grow without mycorrhizas at high P fertilities, which is consistent with both species being facultatively mycotrophic (Janos 2007). Although *A. gerardii* is more dependent upon mycorrhizas than is *E. canadensis*, both plant species are likely to be maximally responsive to mycorrhizas under the P availability conditions of native tallgrass prairies. Even though in our experiments mycorrhizas most improved the P uptake and use efficiency of *E. canadensis* that could have been a consequence of the additional species of AM fungi with which we inoculated it. This underscores that “responsiveness” is influenced not only by plant species P‐use physiology but also by the complement of AM fungus species forming mycorrhizas together with the soil environmental conditions in which they are functioning (Janos 2007).

Although dependence and responsiveness must be determined experimentally, we have shown that they reveal otherwise hidden aspects of plant functional traits with respect to mineral nutrition. For example, if mycorrhiza function is impeded by cold temperature or soil saturation when *E. canadensis* emerges in the early spring, this relatively little‐dependent species can grow without mycorrhizas, especially by favoring root over shoot growth. In contrast, the mycorrhiza‐dependent *A. gerardii* emerges in late spring (Weaver and Fitzpatrick 1934) and may join established common mycorrhizal networks that increase its mycorrhizal colonization (Weremijewicz and Janos 2013). *Andropogon gerardii* allocated proportionally more energy and materials to root production than did *E. canadensis* and that strategy likely helps *A. gerardii* to thrive in hot, dry, late‐summer environments by providing for extensive mycorrhizas. Effects of mycorrhizas on functional traits of *E. canadensis* and *A. gerardii* might contribute to their respective subdominant and dominant status in tallgrass prairies.

## Conflict of Interest

None declared.

## Data Accessibility

All data are presented in the manuscript as tables and figures. Data are available from the Dryad Digital Repository. doi:10.5061/dryad.gt2m0

## Supporting information


**Table S1.** Phosphorus and nitrogen concentrations (%) and N‐to‐P ratios (N:P) of non‐inoculated (Non‐ inoc.) and inoculated (Inoc.) *Andropogon gerardii* and *Elymus canadensis* individuals across a gradient of weekly P concentration additions.
