# Supplementary material for: Mycorrhizas influence functional traits of two tallgrass prairie species
Source: Ecol Evol. 2016 May 17;6(12):3977–90. doi: 10.1002/ece3.2129 (PMC4874859; doi:10.1002/ece3.2129)
Supplement: Supplementary file 1 — Table S1. Phosphorus and nitrogen concentrations (%) and N‐to‐P ratios (N:P) of non‐inoculated (Non‐ inoc.) and inoculated (Inoc.) Andropogon gerardii and Elymus canadensis individuals across a gradient of weekly P concentration additions. [file ECE3-6-3977-s001.docx]

**Supporting Information**

Table S1. Phosphorus and nitrogen concentrations (%) and N-to-P ratios (N:P) of non-inoculated (Non- inoc.) and inoculated (Inoc.) *Andropogon gerardii* and *Elymus canadensis* individuals across a gradient of weekly P concentration additions

|  | *Andropogon gerardii* | | | | | | *Elymus canadensis* | | | | | |
| --- | --- | --- | --- | --- | --- | --- | --- | --- | --- | --- | --- | --- |
|  | Phosphorus (%) | | Nitrogen (%) | | N:P | | Phosphorus (%) | | Nitrogen (%) | | N:P | |
| P  (µg g^-1^) | Non-inoc. | Inoc. | Non-inoc. | Inoc. | Non-inoc. | Inoc. | Non-inoc. | Inoc. | Non-inoc. | Inoc. | Non-inoc. | Inoc. |
| 1 | 0.05 | 0.12 | 1.12 | 1.57 | 23.75 | 12.56 | 0.10 | 0.10 | 1.88 | 2.01 | 19.79 | 19.15 |
| 2 | 0.04 | 0.10 | 1.27 | 1.33 | 28.52 | 13.63 | 0.12 | 0.15 | 1.95 | 1.84 | 15.77 | 12.54 |
| 4 | 0.04 | 0.13 | 1.10 | 1.71 | 26.70 | 13.62 | 0.18 | 0.17 | 1.87 | 1.79 | 10.40 | 10.56 |
| 8 | NA* | NA | NA | NA | NA | NA | 0.27 | 0.26 | 1.94 | 1.86 | 7.10 | 7.22 |
| 16 | 0.10 | 0.15 | 1.28 | 1.25 | 13.50 | 8.40 | 0.33 | 0.31 | 1.84 | 1.82 | 5.56 | 5.86 |
| 32 | 0.17 | 0.30 | 1.38 | 1.53 | 7.95 | 5.02 | 0.39 | 0.41 | 1.80 | 1.81 | 4.65 | 4.44 |
| 64 | 0.22 | 0.27 | 1.22 | 1.48 | 5.61 | 5.45 | 0.42 | 0.42 | 1.83 | 1.72 | 4.37 | 4.14 |
| 128 | 0.27 | 0.23 | 1.15 | 1.32 | 4.25 | 5.64 | 0.49 | 0.42 | 1.85 | 1.63 | 3.78 | 3.86 |

* NA = not available
